# The detection of serum-volatile organic compounds in the diagnostics of hepatocellular carcinoma using gas chromatography–ion mobility spectrometry

**DOI:** 10.3389/fchem.2025.1672220

**Published:** 2025-10-07

**Authors:** Xin Shen, Chenglin Xie, Shijia Zhang, Wen Ai, Ziran Yu, Xu Ye

**Affiliations:** 1 Hunan Cancer Hospital, The Affiliated Cancer Hospital of Xiangya School of Medicine, Central South University, Changsha, China; 2 The Affiliated Hospital of Hunan Academy of Traditional Chinese Medicine, Changsha, China; 3 School of Pharmacy, Hunan University of Chinese Medicine, Changsha, China

**Keywords:** hepatocellular carcinoma, biomarkers, serum, volatile organic compounds, GC-IMS, chemometrics

## Abstract

**Introduction:**

The third most common cause of cancer-related death worldwide is hepatocellular carcinoma (HCC), a malignant liver tumor that usually arises in patients with cirrhosis and chronic liver disease. New biomarkers are required for bidirectional validation of HCC in clinical practice because of its early asymptomatic stage, high mortality rate, and rapid tumor growth, as well as the high rate of false-positive results obtained for the commonly used serum marker alpha-fetoprotein (AFP).

**Methods:**

This study used gas chromatography–ion mobility spectrometry (GC-IMS) combined with chemometrics to analyze the differences in volatile organic compounds (VOCs) in the serum of patients with and without hepatocellular carcinoma (HCC) in order to explore new biomarkers of HCC.

**Results:**

The GC-IMS analysis detected 31 VOCs in serum, including 8 ketones, 11 aldehydes, 6 alcohols, 2 esters, and 1 furans, many of which differed in content between the controls and the patients with HCC.

**Conclusions:**

The VOCs obtained from the use of these analytical devices can serve as a reference for developing low-cost equipment in the future. Considering its high efficiency and low cost, the detection of volatile organic compounds can be used as an auxiliary means of diagnosis or for mass screening of the population.

## 1 Introduction

Hepatocellular carcinoma (HCC) is a malignant liver tumor that commonly arises in patients with chronic liver disease and cirrhosis (Foglia et al., 2023). Its risk factors mainly include viral infections (hepatitis B and C), alcohol consumption, smoking, non-alcoholic fatty liver disease, and exposure to aflatoxin (Taniguchi, 2023; Zhang et al., 2024). It is the third most common cause of cancer-related death worldwide, with high incidence rates in East and Southeast Asia, Southern Europe, and parts of Africa (Nadarevic et al., 2022). It is evident that HCC is an important public health concern, and the diagnosis and monitoring of HCC are particularly crucial.

HCC has no typical symptoms in its early stages, and the tumor progresses rapidly with a high mortality rate (Wang et al., 2024). Currently, most guidelines recommend ultrasonography (US) for early HCC detection (Sharma et al., 2024). The detection sensitivity of US for HCC lesions varies depending on physician experience, ranging from 40% to 80% (Bannaga et al., 2021). Another test that can be used to detect HCC is the serum marker alpha-fetoprotein (AFP), but this test has the drawback of low sensitivity (high false positive results) (Piñero et al., 2020). The sensitivity limit of AFP for clinical HCC diagnosis is 65%, and for preclinical prediction, it is <40% (Luo et al., 2018). HCC can also be diagnosed using non-invasive imaging, such as computed tomography (CT) and magnetic resonance imaging (MRI), which present characteristic imaging features such as arterial hyperexcitability, venous washout, and capsule enhancement (Chan et al., 2024). However, these diagnostic instruments still lack sufficient sensitivity and specificity, and they require tissue biopsy to confirm uncertain HCC lesions detected during scanning. This often leads to a delayed diagnosis, especially in resource-scarce areas (Bannaga et al., 2021).

If HCC is diagnosed at an early stage, surgical interventions such as partial liver resection or liver transplantation can effectively treat it, with a median 5-year survival rate of about 70% (Li and Wang, 2015). Therefore, how to effectively screen populations for HCC using economic, simple, and reliable diagnostic methods to achieve early diagnoses and improve its monitoring and treatment effectiveness is an urgent clinical problem that needs to be solved.

One of the methods currently gaining attention for diagnosing and monitoring cancer is to detect volatile organic compounds (VOCs) in various biological samples, such as exhaled breath, serum, urine, and bile (Daulton et al., 2021; Gui et al., 2023; Tyagi et al., 2021). VOCs are considered to be potential systemic and local biomarkers that can provide unique information on physiological and biochemical processes, indicating an individual’s health status (Liu et al., 2023; Liu et al., 2024). Therefore, various techniques for measuring volatile organic compounds (VOCs)—such as GC-MS (Chapman et al., 2023; Lubes and Goodarzi, 2018), GC-IMS (Al-Difaie et al., 2024; Liu et al., 2024), electronic nose (Wilson and Forse, 2023), and even specially trained canines (Sonoda et al., 2011; Charles et al., 2024)—can be applied to biological samples including exhaled breath, blood, urine, and other bodily fluids for disease detection and prediction (Frerichs et al., 2023).

Serum contains abundant critical biomolecular information; therefore, it plays an important role in the early screening, auxiliary diagnosis, efficacy monitoring, and prognosis evaluation of various cancers. Compared with other biological samples, serum has the following advantages: serum samples can be collected randomly, at regular intervals, or 24 h a day (Zhang et al., 2012). Compared with other biological samples for volatile compound detection, serum has relatively stable biomarkers that are less susceptible to external interference, favoring long-term preservation and repeated testing (Liu et al., 2024). Additionally, serological testing is not affected by recent medication or local gastric lesions and can reflect the patient’s status over time. Serum sampling also has a high acceptance rate among the general population.

Biomarkers in urine may be disrupted by metabolic abnormalities, including stress, alcohol, smoking, certain foods, drugs, and various environmental factors, which can affect the accuracy of test results (Tyagi et al., 2021). Bile collection is challenging and usually requires invasive methods such as surgery or endoscopy, causing patient discomfort and risk. Breath testing may be affected by various factors such as bleeding from peptic ulcers, severe atrophy of gastric mucosa, and the use of medication before testing, potentially yielding false-negative results (Wildner-Christensen et al., 2002). From this, it can be seen that serum biomarker detection, a relatively less invasive and widely accepted detection method compared with other biological sample collection approaches for VOCs detection has the advantages of easy access and reproducibility of detection. This study aimed to investigate the differences in serum VOCs between patients with HCC and patients without HCC, potentially providing evidence for developing a method for early HCC screening.

## 2 Materials and methods

### 2.1 Materials

This study recruited a total of 6 patients with HCC and 2 patients without HCC as controls. The sample numbers for HCC patients are “LC-01 to LC-06,” while the sample numbers for non-HCC patients are NG-01 and NG-02. The research plan was approved by the Ethics Committee of Hunan Cancer Hospital, and informed consent was obtained from all participating patients and their families.

Serum sample collection: All serum samples were obtained from the participants on an empty stomach on the day post-admission. The serum samples were collected in inert, separate procoagulant vessels. The collected samples were centrifuged at 6,000 rpm for 10 min. The supernatant was extracted and stored immediately at −86 °C.

### 2.2 Analysis by GC-IMS

VOCs were analyzed directly via GC-IMS using the FlavorSpec^®^ Gas-Phase Ion Mobility Spectrometer from G.A.S. (Dortmund, Germany).

This study analyzed eight serum samples, comprising six from hepatocellular carcinoma (HCC) patients and two from non-HCC individuals, designated as LC1-6 and NG1-2, respectively. The samples (0.2 mL) were placed in a 20 mL headspace vial, incubated for 20 min at 60 °C, and then 500 µL was injected into the headspace using a non-shunt injection; the vials were rotated at 500 rpm for 20 min (injection needle temperature: 85 °C). Each sample was measured in three parallel groups.

An MXT-WAX capillary column (15 m × 0.53 mm × 1.0 mm, Restek Inc., USA) was used, and the VOCs were maintained at 60 °C with high-purity N_2_ (99.999%) as the carrier gas for 20 min. The flow rate was initially 2.00 mL min^−1^ for 2 min; after 8 min, it was linearly increased to 10.00 mL min^−1^, then 100.00 mL min^−1^ for 10 min, and finally held for 10 min. The runtime was 20 min, and the injection temperature was 80 °C.

IMS conditions: the IMS reagent was 3H (tritium), a 53 mm drift tube was used, the electric field intensity was 500 V cm^−1^, the drift tube temperature was 45 °C, the flow rate of the carrier gas (high-purity N_2_) was 75 mL min^−1^, and the positive ion mode was used.

### 2.3 Statistical analysis

VOCal data processing software (from G.A.S., Dortmund, Germany, version 2.0.0) was used to generate three-dimensional, two-dimensional, differential, and fingerprint spectra of VOCs. Principal component analysis (PCA) was carried out using OmicShare Tools (Mu et al., 2024). TBtools and SIMCA (Version 14.1, Umetrics, Sweden) were used for CA and PLS-DA, respectively.

## 3 Results

### 3.1 Analysis of GC-IMS results

#### 3.1.1 Comparative analysis of VOCs in serum

**FIGURE 1:**
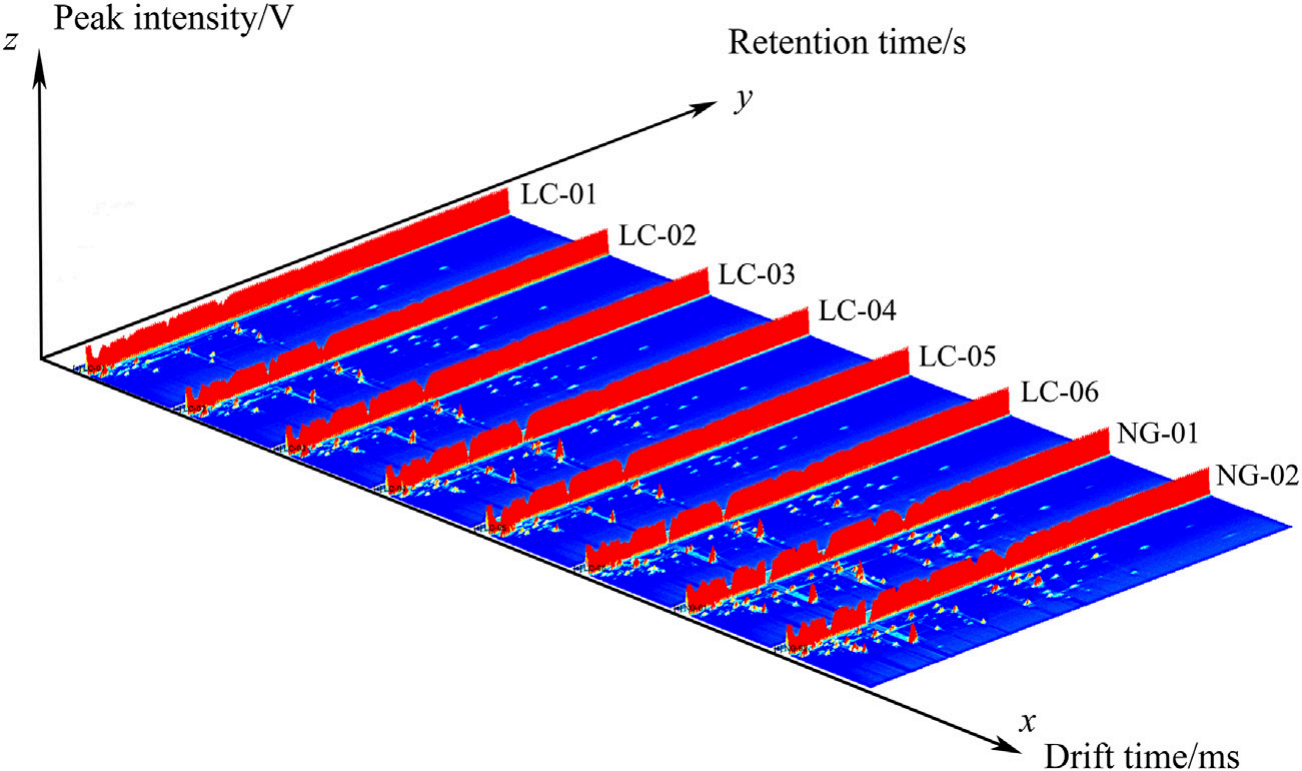
Three-dimensional spectrum of VOCs in the serum samples, analyzed via GC-IMS.

Figure 1 shows the three-dimensional gas-phase ion migration spectrum of VOCs in the serum of patients with and without HCC, generated using the Reporter plugin program in the GC-IMS instrument analysis software. The three coordinate axes represent the relative drift time (X-axis), retention time (Y-axis), and signal peak intensity (Z-axis), respectively. VOCs are represented by peaks, and strength is represented by the height of the red protrusion. A higher red protrusion indicates stronger signals and a higher component content, while a lower red protrusion indicates weaker signals and a lower component content. From Figure 1, it can be seen that there are certain differences in the serum VOCs of patients with HCC relative to those of patients without HCC.

**FIGURE 2:**
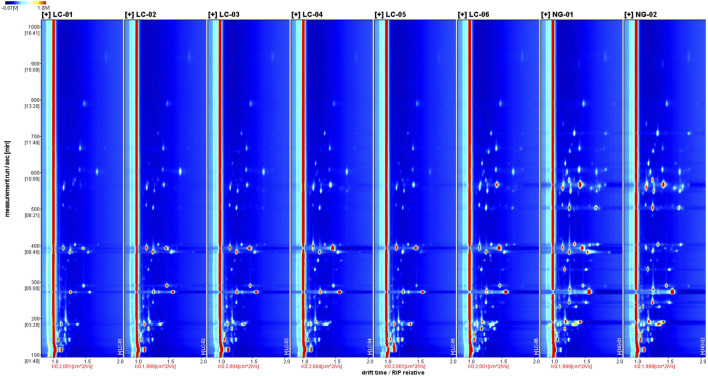
Two-dimensional GC-IMS spectra of VOCs in the serum samples.

Figure 2 shows a two-dimensional top view of volatile compound gas-phase ion migration in different serum samples, with the horizontal axis is a reduced ion mobility and the vertical axis representing retention time. The red vertical line represents the peak of reactant ions, with each bright spot represent in analyte ion intensity. The color and area of the bright spot represent the amount of the volatile compound content. The darker the color and the larger the area of the bright spot, the higher the content of the volatile compound. Red represents a higher content of the corresponding compound, while blue represents a lower content. Figure 2 allows the differences in the volatile compound contents between patients with HCC and patients without HCC to be visually compared.

**FIGURE 3:**
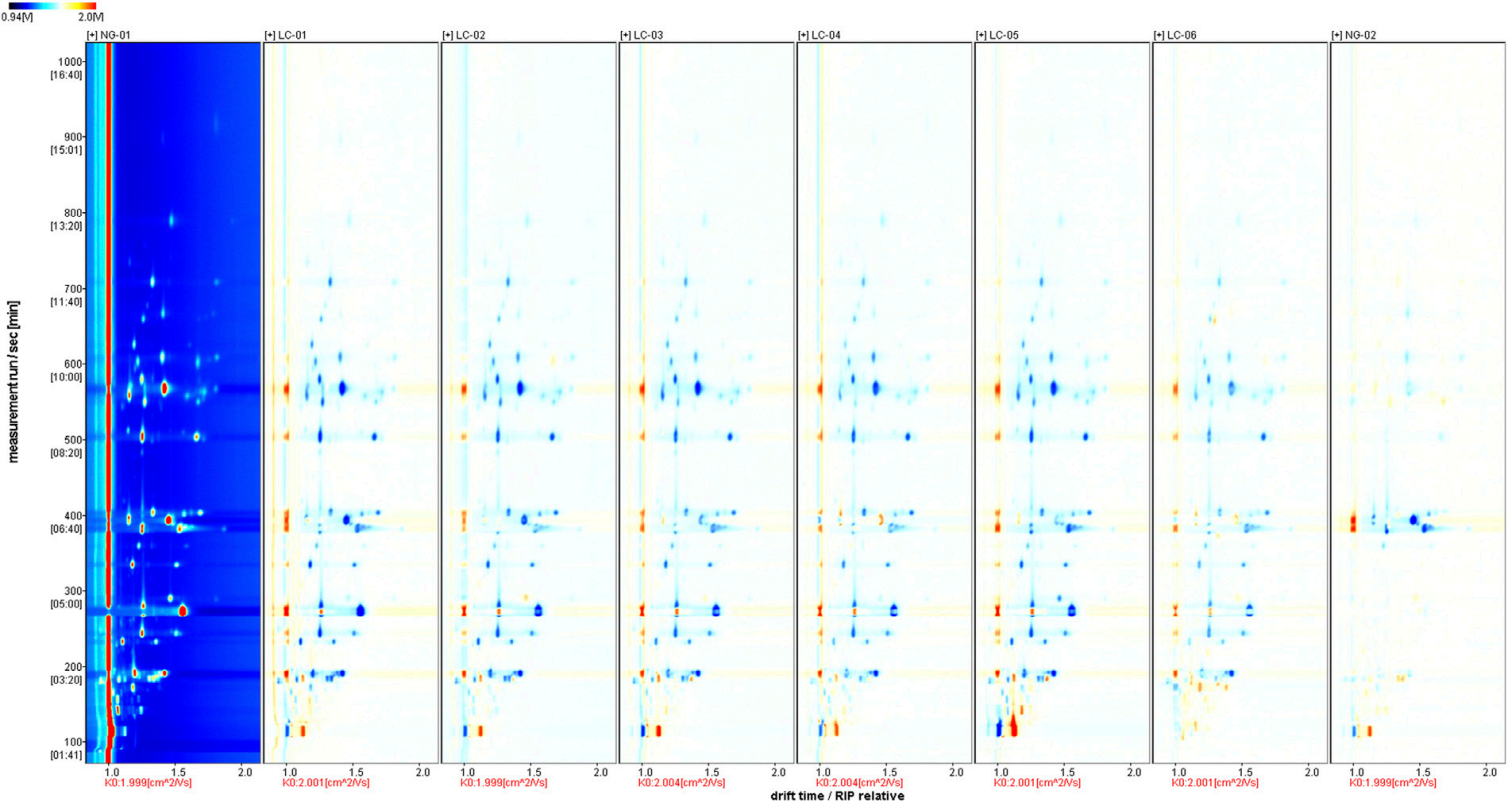
GC-IMS differential spectrum of VOCs using sample NG-01 as the reference.

Figure 3 shows the spectrum of the NC-01 sample selected as a reference. The reason for selecting NC-01 as the reference sample is primarily that the VOCs of serum in patients without HCC represent the baseline level. By comparing these components, we can identify which components in the serum of HCC patients deviate significantly from the normal range in terms of content or proportion, thereby screening out specific biomarkers associated with HCC. This lays the foundation for efficiently identifying differences in VOCs of serum related to HCC. The spectra of the other samples were subtracted from the reference to obtain a comparison chart of the differences between different samples. As long as the volatile organic compound content in the target sample and the reference sample is the same, the subtracted background will be white. In the spectra of other target samples, red indicates that the concentration of a compound in the target sample is higher than the reference concentration, and blue indicates that it is lower. We can clearly see the differences in ionized VOCs signals between patients with HCC and patients without HCC through the differential spectra.

#### 3.1.2 Qualitative analysis

To compare the relative differences of VOCs in the serum samples of patients with and without HCC, the data were processed using a VOCal plugin to obtain the ion migration spectra of the samples. After searching and comparing the GC retention index (NIST 2020) database and IMS drift time database, it was found that a total of 31 VOCs were detected in the samples, including 8 ketones, 11 aldehydes, 6 alcohols, 2 esters, and 1 furans. The qualitative results are shown in Table 1.

**TABLE 1:** The VOCs detected in the samples.

Compound	Comment	CAS	Molecular formula	MW	RI	Rt/s	IMS-related ions	Dt/ms
Acetone	-	67-64-1	C_3_H_6_O	58.1	551.7	123.028	C_3_H_7_O^+^	1.11685
Ethanol	-	64-17-5	C_2_H_6_O	46.1	529.3	114.318	C_2_H_7_O^+^	1.12843
2-Butanone	2-Butanone-M	78-93-3	C_4_H_8_O	72.1	596.9	142.124	C_4_H_9_O^+^	1.06706
	2-Butanone-D						(C_4_H_8_O)_2_H^+^	1.24884
Acetic acid ethyl ester	-	141-78-6	C_4_H_8_O_2_	88.1	651.9	165.575	C_4_H_9_O_2_^+^	1.09601
Pentanal	Pentanal-M	110-62-3	C_5_H_10_O	86.1	700.2	191.371	C_5_H_11_O^+^	1.20021
	Pentanal-D						(C_5_H_10_O)_2_H^+^	1.42482
1-Butanol	1-Butanol-M	71-36-3	C_4_H_10_O	74.1	666.2	172.275	C_4_H_11_O^+^	1.18516
	1-Butanol-D						(C_4_H_10_O)_2_H^+^	1.38430
1-Penten-3-ol	-	616-25-1	C_5_H_10_O	86.1	683.9	180.986	C_5_H_11_O^+^	0.94086
1-Penten-3-one	1-Penten-3-one-M	1629-58-9	C_5_H_8_O	84.1	690.5	184.671	C_5_H_9_O^+^	1.07980
	1-Penten-3-one-D						(C_5_H_8_O)_2_H^+^	1.30789
2-Pentanone	2-Pentanone-M	107-87-9	C_5_H_10_O	86.1	691.0	185.006	C_5_H_11_O^+^	1.12495
	2-Pentanone-D						(C_5_H_10_O)_2_H^+^	1.37388
3-(Methylthio)-1-propene	-	10152-76-8	C_4_H_8_S	88.2	702.6	193.046	C_4_H_9_S^+^	1.04506
(Z)-2-Penten-1-ol	-	1576-95-0	C_5_H_10_O	86.1	771.9	249.329	C_5_H_11_O^+^	0.94202
(E)-2-Pentenal	(E)-2-Pentenal-M	1576-87-0	C_5_H_8_O	84.1	754.2	233.583	C_5_H_9_O^+^	1.10759
	(E)-2-Pentenal-D						(C_5_H_8_O)_2_H^+^	1.35883
1-Pentanol	1-Pentanol-M	71-41-0	C_5_H_12_O	88.1	766.0	243.968	C_5_H_13_O^+^	1.25231
	1-Pentanol-D						(C_5_H_12_O)_2_H^+^	1.51166
Hexanal	Hexanal-M	66-25-1	C_6_H_12_O	100.2	796.7	272.780	C_6_H_13_O^+^	1.25926
	Hexanal-D						(C_6_H_12_O)_2_H^+^	1.56409
2-Hexenal	2-Hexenal-M	505-57-7	C_6_H_10_O	98.1	854.5	334.828	C_6_H_11_O^+^	1.18161
	2-Hexenal-D						(C_6_H_10_O)_2_H^+^	1.51852
4-Heptanone	4-Heptanone-M	123-19-3	C_7_H_14_O	114.2	875.6	360.834	C_7_H_15_O^+^	1.23532
	4-Heptanone-D						(C_7_H_14_O)_2_H^+^	1.59013
Cyclohexanone	Cyclohexanone-M	108-94-1	C_6_H_10_O	98.1	899.6	393.775	C_6_H_11_O^+^	1.15068
	Cyclohexanone-D						(C_6_H_10_O)_2_H^+^	1.45992
Heptanal	Heptanal-M	111-71-7	C_7_H_14_O	114.2	906.3	404.177	C_7_H_15_O^+^	1.33460
	Heptanal-D						(C_7_H_14_O)_2_H^+^	1.69429
(E)-2-Heptenal	(E)-2-Heptenal-M	18829-55-5	C_7_H_12_O	112.2	962.5	502.683	C_7_H_13_O^+^	1.25532
	(E)-2-Heptenal-D						(C_7_H_12_O)_2_H^+^	1.66253
Benzaldehyde	-	100-52-7	C_7_H_6_O	106.1	966.9	511.417	C_7_H_7_O	1.15061
1-Octen-3-one	1-Octen-3-one-M	4312-99-6	C_8_H_14_O	126.2	985.7	550.096	C_8_H_15_O^+^	1.27859
	1-Octen-3-one-D						(C_8_H_14_O)_2_H^+^	1.67804
n-Octanal	n-Octanal-M	124-13-0	C_8_H_16_O	128.2	1018.6 1018	610.720	C_8_H_17_O^+^	1.40613
	n-Octanal-D					609.615	(C_8_H_16_O)_2_H^+^	1.82434
2-Pentyl furan	-	3777-69-3	C9H_14_O	138.2	999.9	579.233	C9H_15_O^+^	1.25285
1-Octen-3-ol	1-Octen-3-ol-M	3391-86-4	C_8_H_16_O	128.2	990.3	559.899	C_8_H_17_O^+^	1.16012
	1-Octen-3-ol-D						(C_8_H_16_O)_2_H^+^	1.59725
(E)-2-Octenal	(E)-2-Octenal-M	2548-87-0	C_8_H_14_O	126.2	1070.9	708.495	C_8_H_15_O^+^	1.33043
	(E)-2-Octenal-D						(C_8_H_14_O)_2_H^+^	1.82244
2-phenylacetaldehyde	-	122-78-1	C_8_H_8_O	120.2	1046.2	660.436	C_8_H_9_O^+^	1.26042
(E,E)-2,4-Heptadienal	(E,E)-2,4-Heptadienal-M	4313-03-5	C_7_H_10_O	110.2	1027.4	626.187	C_7_H_11_O^+^	1.19608
	(E,E)-2,4-Heptadienal-D						(C_7_H_10_O)_2_H^+^	1.61807
n-Nonanal	-	124-19-6	C_9_H_18_O	142.2	1109.1	789.582	C_9_H_19_O^+^	1.47504
Tetrahydrofurane	Tetrahydrofurane-M	109-99-9	C_4_H_8_O	72.1	633.5	157.307	C_4_H_9_O^+^	1.06597
	Tetrahydrofurane-D						(C_4_H_8_O)_2_H^+^	1.22493
Ethyl 2-methylpropanoate	-	97-62-1	C_6_H_12_O_2_	116.2	761.3	239.801	C_6_H_13_O_2_^+^	1.18898
3-Hexanone	-	589-38-8	C_6_H_12_O	100.2	784.1	260.850	C_6_H_13_O^+^	1.17194

The substance suffixes M and D represent monomers and dimers of the same substance, RI is the retention index, Rt is the retention time, Dt is the drift time.

#### 3.1.3 GC-IMS fingerprint analysis

**FIGURE 4:**
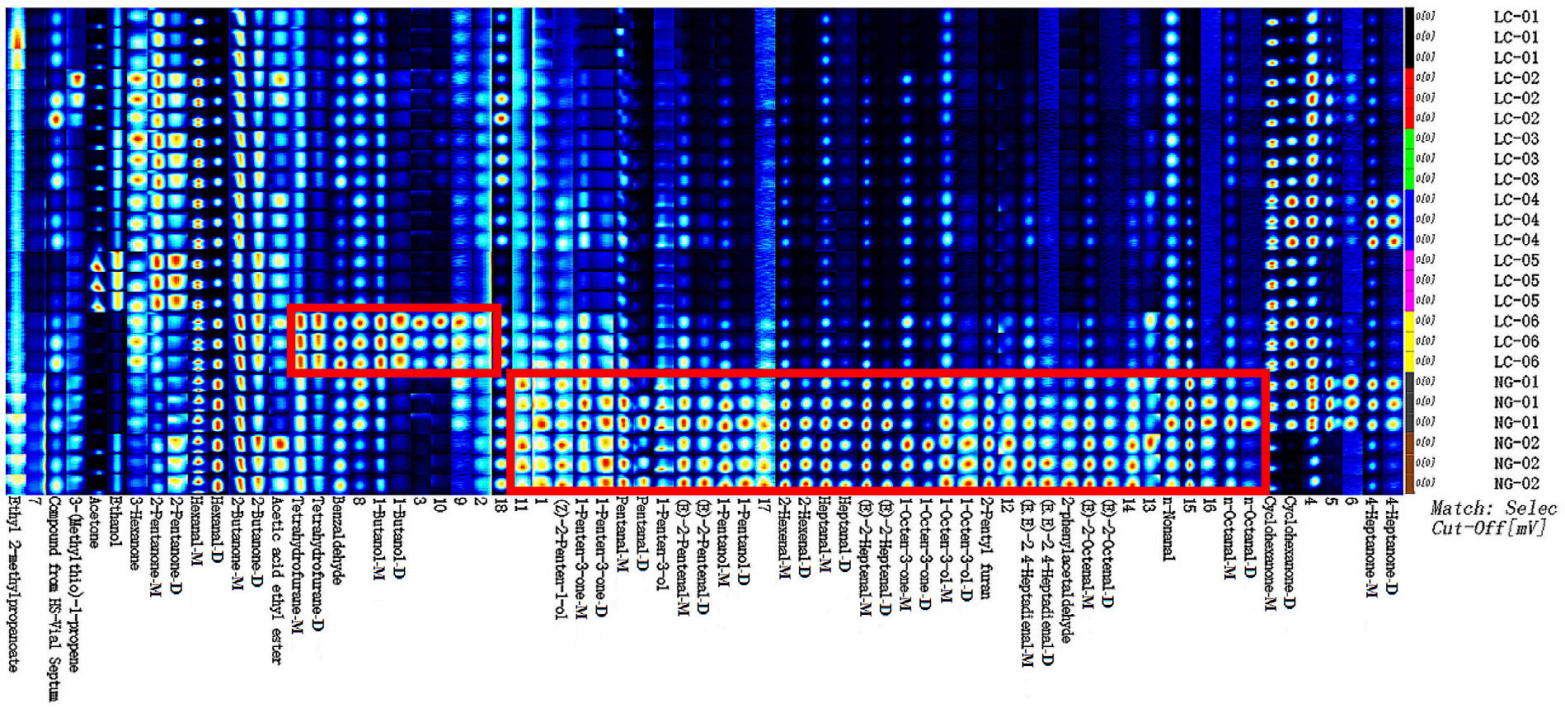
Fingerprint of VOCs in the serum samples.

To further compare the VOCs in different serum samples, fingerprint analysis was performed on all VOCs, and the results are shown in Figure 4. This Figure shows all selected signal peaks from a sample in rows, and each volatile organic compound’s signal peak in columns. A grid shows a higher content if the color is brighter. Through comparative analysis of the volatile substances detected in different serum samples, it was found that there are significant differences in the VOCs detected in the serum of patients with and without HCC. The results indicate differences in the content of n-Octanal, n-Nonanal, (E)-2-Octenal, 2-phenylacetaldehyde, (E.E)-2.4-Heptadienal, 2-Pentyl furan, 1-Octen-3-ol, 1-Octen-3-one, (E)-2-Heptenal, Heptanal, 2-Hexenal, 1-Pentanol, (E)-2-Pentenal, 1-Penten-3-ol, Pentanal, 1-Penten-3-one and (Z)-2-Penten-1-ol. Their contents are all higher in NG-01 and NG-02; that is, their contents are higher in the serum of patients without HCC and are very low in the serum of patients with HCC. The contents of tetrahydrofuran, Benzaldehyde, and 1-Butanol were higher in the LC-06 sample, while they were very low in the other patients with HCC and in patients without HCC.

### 3.2 Chemometrics

#### 3.2.1 Principal component analysis (PCA)

**FIGURE 5:**
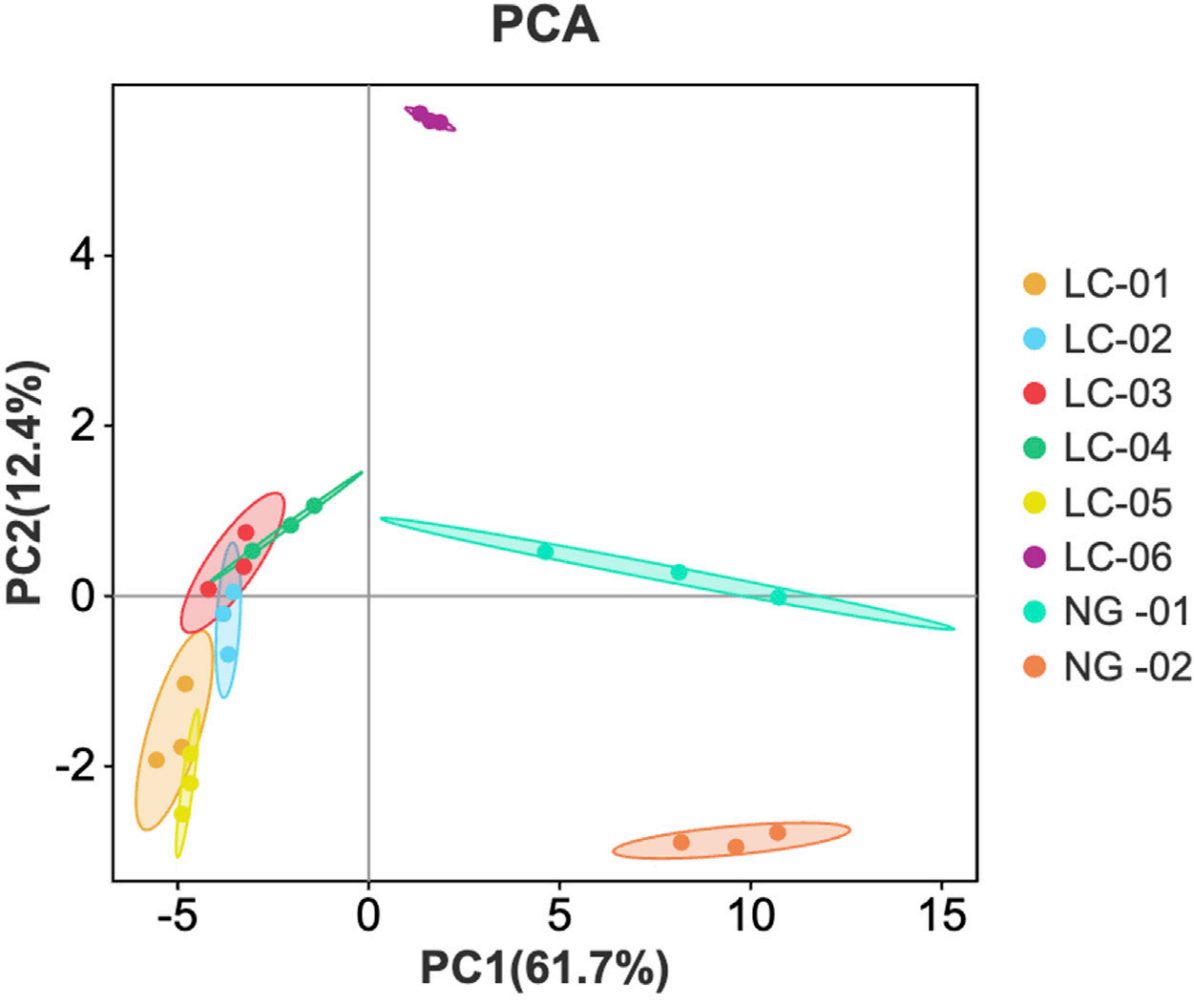
PCA score plot of VOCs in the serum samples.

PCA is a data representation method used for feature extraction and dimensionality reduction. This method converts many potentially related variables into a small number of unrelated variables, providing an overview of category separation, clustering, and outliers. PCA visualizes the model, making it easy to understand and avoiding subjective judgments. PCA of the VOCs detected in the serum samples from patients with and without HCC was performed using Origin 2024b software for LC-01, LC-02, LC-03, LC-04, LC-05, LC-06, NG-01, and NG-02. The results are shown in Figure 5. The contribution rate of PC1 is 61.7%, the contribution rate of PC2 is 12.4%, and the cumulative contribution rate reaches 74.1%. The distances between samples LC-01, LC-02, LC-03, LC-04, and LC-05 are relatively small, indicating that their differences are small. However, there is a significant gap between the six LC groups and the two NG groups, indicating that there is a large difference in serum VOCs between patients with HCC and patients without HCC. The serum VOCs of patients with HCC and patients without HCC were classified using PCA, confirming that there is a significant difference in serum VOCs between these two groups of patients. The LC-06 contains some different VOCs, which may be related to its comorbidities.

#### 3.2.2 Partial least-squares discriminant analysis (PLS-DA)

**FIGURE 6:**
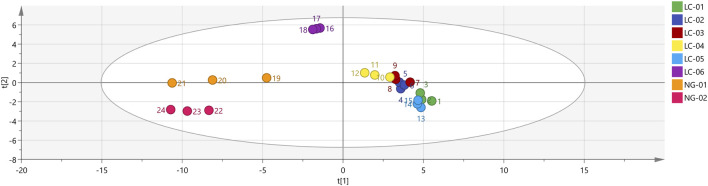
PLS−DA analysis of VOCs in eight groups of serum.

**FIGURE 7:**
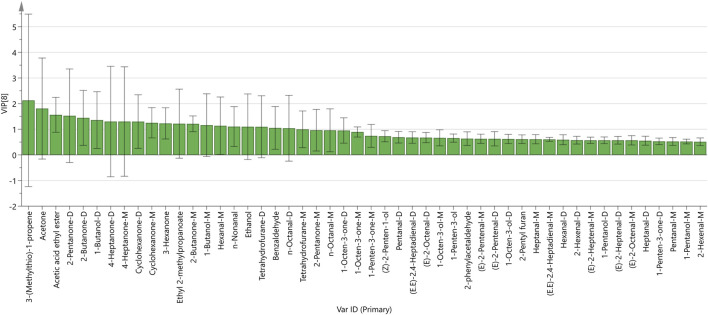
VIP values of the characteristic variables.

**FIGURE 8:**
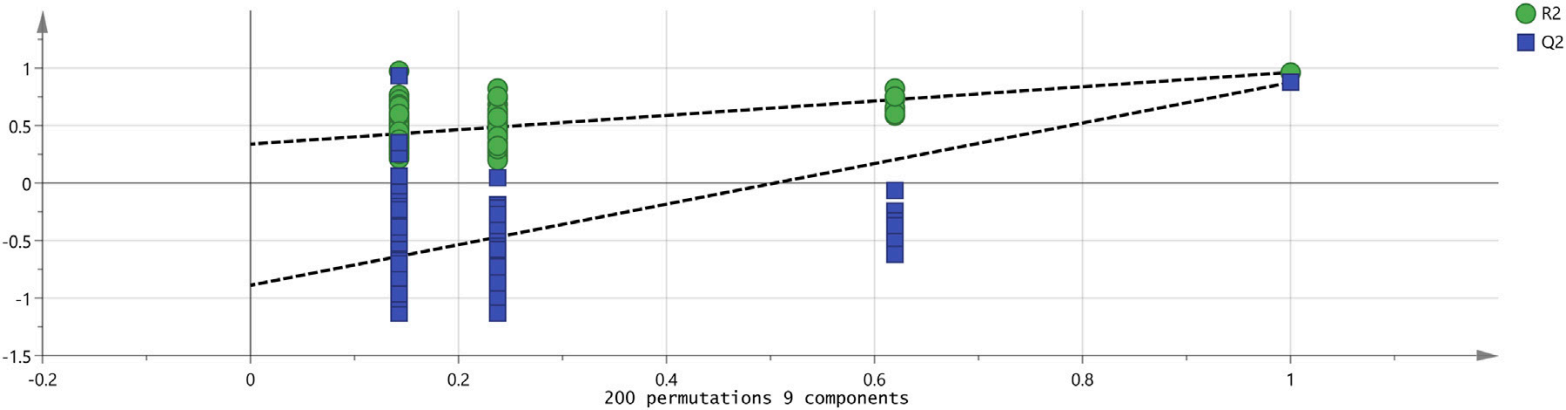
Permutation test results of VOCs in eight groups of serum.

To better observe the differences between groups, a PLS-DA model was established for patients with HCC and patients without HCC. PLS-DA is a statistical method for supervised discriminant analysis that is different from PCA. It can interpret observations and make predictions for corresponding variables. Eight sets of sample data were imported into SIMCA software for analysis, and the results are shown in Figure 6. In the PLS-DA model, R^2^ and Q^2^ are used to evaluate the model reliability and predictive ability, respectively. Values of R^2^ and Q^2^ greater than 0.5 indicate acceptable model fitting, while a value close to 1 indicates strong predictive ability. The PLS-DA scoring chart shows that the PLS-DA model has good predictive ability (R^2^X = 0.987, R^2^Y = 0.976, Q^2^ = 0.899). The values of R^2^ and Q^2^ are close to 1, indicating good fitting accuracy. LC-01, LC-02, LC-03, LC-04, and LC-05 are distributed on the right side of the graph, while NG-01 and NG-02 are on the left side of the graph, presenting distant clusters and indicating significant differences in VOCs in the serum of patients with HCC and patients without HCC; these findings are consistent with the conclusion drawn from the PCA graph. In addition, the predicted variable importance in projection (VIP)>1 is used as a standard to measure the strength and explanatory power of the expression pattern of the compounds for the classification discrimination of each group of samples, assisting in the screening of the main distinguishing peaks between samples. As shown in Figure 7, 3-(Methylthio)-1-propene, Acetone, Acetic acid ethyl ester, 2-Pentanone, 2-Butanone, 1-Butanol, 4-Heptanone, 4-Heptanone, Cyclohexanone, Ethyl 2-methylpropanate, Cyclohexanone, 2-Butanone, n-Nonanal, 3-Hexanone, 1-Butanol, n-Octanal, Hexanal, Tetrahydrofuran, Ethanol, n-Octanal, and Benzaldehydryl are the main indicator components of the difference. To determine whether the model is overfit, we conducted 200 cross-validations to examine the R^2^ and Q^2^ values. From the graph, we observed that the slope of the line is large, indicating that the PLS-DA model is not overfit (R^2^ = 0.339, Q^2^ = −0.889; as shown in Figure 8.

## 4 Discussion

This study utilized GC-IMS combined with chemometrics to analyze the effects of serum VOCs in patients with and without HCC. A total of 31 VOCs were detected, including 8 ketones, 11 aldehydes, 6 alcohols, 2 esters, and 1 furans. By using GC-IMS technology to obtain three-dimensional, two-dimensional, and color difference fingerprint spectra, we can preliminarily showed/could potentially visualize significant differences in the volatile compounds detected in the serum of patients with HCC and patients without HCC.

According to the results of the volatile compound fingerprint spectrum, it can be concluded that one of the patients with HCC (LC-06) exhibits unique specificity in serum VOCs, mainly featuring a higher content of Tetrahydrofuran, Benzaldehyde, and 1-Butanol, while their contents are very low in the patients in the other HCC groups and patients without HCC. Among these substances, the PLS-DA model, established using SIMCA, shows that the substances with VIP values greater than 1 are Tetrahydrofuran, Benzaldehyde, and 1-Butanol.

The content of n-Octanal, n-Nonanal, (E)-2-Octenal, 2-phenylacetaldehyde, (E.E)-2.4-Heptadienal, 2-Pentyl furan, 1-Octen-3-ol, 1-Octen-3-one, (E)-2-Heptenal, Heptanal, 2-Hexenal, 1-Pentanol, (E)-2-Pentenal, 1-Penten-3-ol, Pentanal, 1-Penten-3-one, and (Z)-2-Penten-1-ol were higher in the serum of patients without HCC and very low in the serum of patients with HCC. Most of these substances are aldehydes and alcohols. The PLS-DA model shows that the substances with VIP values greater than 1 are n-nonanal.

The VIP evaluation conducted on the eight groups of serum samples in this study significantly reduced data complexity and improved the classification and diagnostic performance of the discriminant model. In this study, VIP evaluation was used to exclude over 90% of the features from the discriminative model, resulting in better classification results. This highlights the importance of variable selection for model performance. Including a large number of variables in a discriminative model may prove ineffective and counterproductive, while selecting a smaller number of variables with significant correlations can lead to better performance. The use of appropriate stoichiometric data processing, combined with analytical methods that provide a wealth of information, has been proven to be decisive in achieving satisfactory results (Rodríguez-Hernández et al., 2023).

Serum VOCs testing, with its advantages of high efficiency and low cost, has the potential to become a promising method for understanding diseases through the development of reliable, sensitive, and reproducible diagnostic tests. After many years of exploration, the research on serum biomarkers has yielded fruitful results, and the analysis of many material indicators has greatly advanced the progress of clinical diagnosis of tumors (Liu et al., 2023; Liu et al., 2024). However, in-depth exploration of the diagnostic value of VOCs in the serum of cancer patients has been lacking. The detection of serum VOC markers, has a high acceptance rate among patients and the advantages of easy access and repeatability. It has become a new approach for tumor diagnosis, especially early diagnosis. However, its effectiveness still needs to be confirmed with further validation studies.

In recent years, gas chromatography-ion mobility spectrometry (GC-IMS) has shown unique advantages as a new analytical method for the detection and analysis of volatile organic compounds (Pan, et al., 2024). The results obtained using GC-IMS show its significance as an analytical tool, as this method is much simpler than using high-end analytical methods such as GC-MS and does not require a laboratory environment. At the same time, GC-IMS combines the excellent separation performance of gas chromatography with the high sensitivity of ion mobility spectrometry, and can be created in a portable form, using nitrogen or air as the carrier gas (Dai et al., 2024; Tyagi et al., 2021; Xiang et al., 2023). It can accurately analyze VOCs without the need for tedious sample pretreatment, greatly simplifying the analysis process (Zheng et al., 2024). This innovative method is not only expected to significantly improve diagnostic efficiency, but also significantly reduce the cost of diagnosis and treatment for patients. Furthermore, its minimally invasive nature can effectively improve patient cooperation and bring more convenience to clinical diagnosis. In the future, detecting serum VOCs in combination with other screening methods can enhance diagnostic performance and deepen the analysis of VOCs and their metabolic pathways, opening new paths for tumor diagnosis and treatment. We believe that the use of serum VOCs will be an important diagnostic tool in the future.

## 5 Limitations

The limitation of this study is that the sample size is too small. These results will need to be further verified in the clinic.

## 6 Conclusion

In this study, GC-IMS combined with chemometrics was utilized to analyse and compare the VOCs detected in the serum samples of patients with and without HCC. The results showed that there were significant differences in VOCs in the serum samples of patients with HCC and patients without HCC. Some VOCs were reduced in patients with HCC, mostly aldehydes and alcohols. The VOCs obtained from the use of these analytical devices can serve as a reference for developing low-cost equipment in the future. Considering its high efficiency and low cost, the detection of volatile organic compounds can be used as an auxiliary means of diagnosis or for mass screening of the population.

## Data Availability

The original contributions presented in the study are included in the article/supplementary material, further inquiries can be directed to the corresponding author.
